# A universal route with fine kinetic control to a family of penta-twinned gold nanocrystals†

**DOI:** 10.1039/d1sc03040j

**Published:** 2021-08-24

**Authors:** Tao Zhang, Xuejiao Li, Yiqiang Sun, Dilong Liu, Cuncheng Li, Weiping Cai, Yue Li

**Affiliations:** Key Laboratory of Materials Physics, Institute of Solid State Physics, HFIPS, Chinese Academy of Sciences Hefei 230031 China yueli@issp.ac.cn; School of Chemistry and Chemical Engineering, University of Jinan Jinan 250022 Shandong P. R. China; University of Science and Technology of China Hefei 230026 P. R. China

## Abstract

Some of the major difficulties hindering the synthesis of different types of colloidal nanocrystals are their complex synthetic methods and the lack of a universal growth mechanism in one system. Herein, we propose a general strategy of kinetically controlled seed-mediated growth to synthesize a family of penta-twinned gold nanocrystals. Specifically, different kinds of penta-twinned nanocrystals (truncated penta-twinned decahedra, truncated bipyramids, bipyramids, truncated bipyramids with tips, star-like penta-twinned nanocrystals, decahedra with concave edges, and decahedra) with tunable sizes and high purity were readily achieved in one system solely by tailoring the deposition kinetics of adatoms on different sites of decahedral seeds. The controllable deposition kinetics can be realized by changing the ratio of reductant/gold precursors (*R*), which dictates whether horizontal or vertical features along the 5-fold axis direction of Au decahedral seeds are produced. Additionally, the selective growth of a second metal (silver) on penta-twinned gold seeds can be reached through minor modification of *R*, which opens a new avenue for mechanistic investigation by visualizing the seed localization within the final particles. The present work demonstrates a general paradigm for the kinetic growth of penta-twinned crystals and would be extended to the synthesis of other families of nanocrystals.

## Abstract 2

We report the synthesis of linear ADPr oligomers of defined length up to a pentamer using an improved solid phase method. Binding study with human oncogenic helicase ALC1 shows that ADPr oligomers bind to ALC1 in a length-dependent manner.

## Graphical abstract

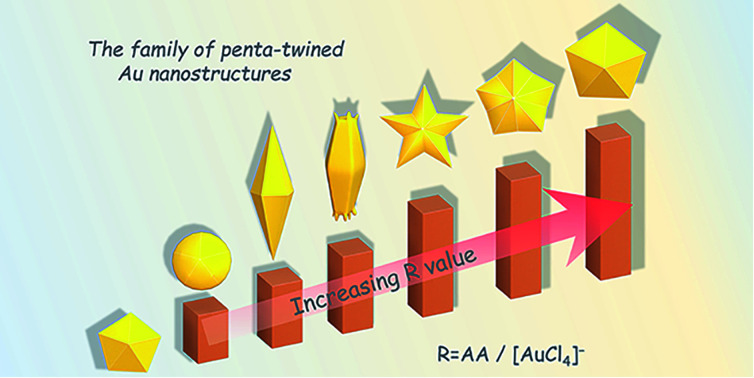

## 1 Introduction

Crystallographic twins usually form when two crystals connect *via* the same crystal interface and intergrow in a certain direction.^1–3^ Due to their unique physical and chemical properties caused by strain, multiple twinned nanomaterials have attracted wide attention in crystal growth,^4^ biomedical diagnosis,^5^ optics,^6^ and catalysis.^7,8^ Notably, the family of penta-twinned gold nanocrystals (Au NCs) comprises some of the most interesting NCs, which have 5-fold symmetry, well-defined twinned structures, and controllable aspect ratios; these NCs also have interesting plasmonic diversity that involves a broad optical region from the visible to near-infrared (NIR) region.^9–12^ Geometrically, a basic decahedron (DH) is composed of five tetrahedral blocks equal-angularly separated by twin boundaries, and the five blocks share the 5-fold axis of the NC.^13^ The other penta-twinned Au NCs, such as penta-twinned bipyramids (BPs), truncated BPs, and nanorods (NRs), elongate or widen along the vertical or horizontal direction of the 5-fold axis.^14–16^ To sufficiently harness their shape- and size-based plasmonic properties and simplify the fabrication process, discovering a general growth method in one system is of primary importance to achieve a generic growth mechanism and reproducible preparation.

Penta-twinned Au NCs are generally prepared *via* a seeded growth procedure, in which small penta-twinned seeds are first prepared and then grown into final products. Both the seed quality and growth environment thus have a critical influence on their synthesis. In early research, their shape yield was generally less than 30% because of the poor portion of penta-twinned seeds used in the original synthesis.^10^ Therefore, time-consuming purification steps of Au decahedral seeds or final products were usually needed.^17–19^ Originally, the Parola and Liz-Marzan groups reported that the seed thermal treatment enables the conversion of seeds into much more stable penta-twinned ones, dramatically increasing the yield of products to 90% without purification.^16,20^ Recently, a new breakthrough in the formation mechanism of penta-twinned seeds was discovered by *in situ* high-resolution transmission electron microscopy, revealing their real formation process.^1^ Now, we can obtain a comprehensive understanding and control of decahedral seed formation based on the above results.

In addition to the seed quality, the growth conditions are another factor affecting the synthesis of penta-twinned NCs. In fact, the same penta-twinned seeds can evolve into different shapes with the same 5-fold symmetry by different methods.^15,21,22^ In the mainstream synthesis of Au penta-twinned NCs, the aqueous growth solution mainly contains chloroauric acid, surfactants (CTAB, CTAC, DBAC, CTEAB, *etc.*), reductants (hydroquinone, ascorbic acid, *etc.*), and other additives (Ag^+^, Pt^2+^, *etc.*).^23–26^ Due to the formation of dendrites by recrystallization at a lower temperature, the CTA^+^-capped Au BPs or NRs as NIR-active nanomaterials must undergo complex pretreatments when used in biomedicine.^27–29^ The role of foreign metal ions is to preferentially absorb onto a given surface of NCs in the form of atomic monolayers by underpotential deposition.^14,19,30^ It should be noted that these introduced surfactants and foreign ions can facilitate the exposure of a specific plane by thermodynamic control.^31–33^ In view of thermodynamics, it is not favorable to fabricate sharp tips that are related to the products of site-selected growth.^34^ Considering the complexity of different kinds of surfactants, the concentration of foreign metal ions, and the combination of several parameters, it is very difficult to unravel the underlying universal mechanism and achieve the desired reproducibility. In fact, NCs with different nanostructures can be accessed through kinetic control, in which adatoms preferentially deposit onto the given sites of seeds with higher surface energy.^35,36^ Inspired by this discovery, we believe kinetically-controlled seeded growth may promote the diversity of penta-twinned NCs in one system and lead to a clearer understanding of a universal growth mechanism by avoiding complex changes in surfactants and foreign metal ions. However, this goal still presents great challenges due to the lack of fine kinetic control of growth.

Here, we develop a general synthesis strategy, kinetically-controlled seed-mediated growth, to prepare a family of Au penta-twinned NCs in one growth system by modulating atom deposition sites through tailoring the *R* value (the molar ratio of reductant/Au precursors). We further leverage this ability to tune different kinetic regimes, providing access to seven types of penta-twinned NCs with well-defined size, shape, or aspect ratio in a high yield (>95%). Through quantitative analysis of their growth kinetics, a universal mechanism toward the formation of the penta-twinned NCs is presented. This proposed method can be further applied to the growth of a second metal (silver) on Au decahedral seeds and enable the visualization of seeds in the final products. This presented route does not require any pretreatments (thermally treated seeds or purification of products) and additional metal ions, and the prepared penta-twinned NCs will have potential applications in different fields. The general strategy demonstrated here highlights a new kinetically controlled strategy for the family of penta-twinned NCs and consummates a universal mechanism for their formation, which may realize selective mass production in a designed growth mode.

## 2 Experimental section

### 2.1 Materials

Chloroauric acid (HAuCl_4_, 99.9%) and poly(diallyldimethylammonium)chloride (PDDA, *M*_w_ = 100 000–200 000) were obtained from Sigma-Aldrich. Ascorbic acid (AA, ≥99.8%), ethylene glycol (ACS reagent), and silver nitrate (AgNO_3_, ≥99.8%) were purchased from Sinopharm Chemical Reagent Co., Ltd. Purified water with a resistivity of 18.25 MΩ was prepared using a Milli-Q water treatment instrument.

### 2.2 Synthesis of Au decahedral seeds

In a standard synthesis, given amounts of HAuCl_4_, PDDA and AgNO_3_ were added to 70 mL of EG solution. The final concentrations of HAuCl_4_, PDDA, and AgNO_3_ in the solution were about 0.25, 25, and 1.2 mM, respectively. Then, the mixed solution was stirred until a homogeneous mixture was formed, followed by heating in an oil bath at 220 °C for 5 h. Finally, the Au colloidal solution was transferred into another bottle without shape purification procedures. The residual AgCl precipitates at the bottom of the initial reaction bottle were removed. The final products were collected by centrifugation and washed repeatedly with deionized water for characterization. Au decahedral seeds with average edge lengths of 19.1 ± 1.2, 23.3 ± 2.0, 25.0 ± 2.2, 28.5 ± 1.8, 32.2 ± 1.1, 38.7 ± 1.3, and 49.6 ± 1.8 nm could be obtained by only changing the reaction temperature to 220, 218, 215, 210, 204, 200, and 185 °C, respectively. Note: the centrifugation step is not a shape selection procedure, and it was just used to remove the residual solvent and surfactants.

### 2.3 Synthesis of Au BPs

In a standard synthesis, 0.07 mL of 1 M HAuCl_4_ and 0.28 mL of 0.5 M AA aqueous solution were added to 70 mL of the as-prepared Au decahedral seed solution in a glass vial. Specifically, we set the molar ratio of AA and HAuCl_4_ as *R*. The mixture was shaken to form a homogeneous colloidal solution. The concentrations of HAuCl_4_, PDDA, and AA were 1, 25, and 2 mM (*R* = 2), respectively. Subsequently, the solution mixture was kept in an oven at 50 °C for 2 h to obtain a brown colloidal solution. The Au decahedra were collected by centrifugation with DI water 3 times for further use. No shape purification was involved in this synthesis process.

### 2.4 Synthesis of star-like Au NCs

In a typical synthesis, 0.035 mL of 1 M HAuCl_4_ and 0.7 mL of 0.5 M AA aqueous solution were added to 70 mL of Au decahedral seed solution. The concentrations of HAuCl_4_, PDDA, and AA were 0.5, 25, and 5 mM (*R* = 10), respectively. Subsequently, the solution mixture was kept in an oven at 50 °C for 1 h to get a dark blue colloidal solution. The star-like Au NCs were collected by centrifugation with DI water 3 times for further use. Note: star-like Au NCs can be synthesized in a wide *R* value range (8 < *R* ≤ 20).

The synthesis methods of truncated penta-twinned decahedra, truncated Au BPs, truncated Au BPs with tips, and decahedra with larger size are the same as that of star-like Au NCs, except for the *R* value. It means that the shape of the final products can be controlled by *R* value. Specifically, truncated penta-twinned Au decahedra (1 ≤ *R* ≤ 1.4), truncated Au BPs (1.4 < *R* ≤ 2), truncated Au BPs with tips (2 ≤ *R* ≤ 7), decahedra with concave edges (20 < *R* < 25), and decahedra with larger size (25 ≤ *R* ≤ 100) were synthesized with the given *R* value range. The Au products were collected by centrifugation with DI water 3 times for further use. No shape purification was involved in all the above synthesis methods. All the TEM images were obtained from the samples of Au NCs without any purification.

### 2.5 Synthesis of Au@Ag nanorods and decahedra

Briefly, given amounts of AgNO_3_ and AA aqueous solution were added to 70 mL of Au decahedral seed solution in a glass vial. Specifically, we set the molar ratio of AA and AgNO_3_ as *R*. The concentrations of AgNO_3_, PDDA, and AA were set as 2, 25, and 4 mM (*R* = 2), respectively. Subsequently, the solution mixture was kept in an oven at 50 °C for 2 h to obtain a dark yellow colloidal solution. To obtain Au@Ag NRs with different aspect ratios, the concentration of AgNO_3_ can be increased from 1 to 5 mM, while keeping the *R* value as 2. The synthesis of Au@Ag decahedra is similar to that of Au@Ag nanorods except that *R* = 50. No shape purification was involved.

### 2.6 Instruments

The morphology of all the as-prepared products was characterized by field emission scanning electron microscopy (FE-SEM, SIGMA 500) and transmission electron microscopy (TEM, JEOL, JEM-1400). The EDS, HRTEM, and HAADF-STEM images were obtained on a Tecnai G2 F20. For their optical property measurements, these products were redispersed in water homogeneously and analyzed using a Shimadzu UV-3101PC spectrophotometer.

## 3 Results and discussion

### 3.1 Kinetic control of seeded growth

In this study, Au decahedral seeds were first synthesized by polyol reduction using ethylene glycol (EG) and poly(diallyldimethylammonium)chloride (PDDA) as the reductant and surfactant, respectively (Fig. S1†). Compared to the CTA^+^-capped Au seeds, the PDDA-capped Au decahedral seeds presented in this method were formed in high yield (>95%), and avoided the need of seed purification or thermal treatment before regrowth (Fig. S1a and b†). The atomic percentage of Ag in Au decahedra is about 6% as evaluated by energy dispersive X-ray spectroscopy (EDS), shown in Fig. S1e.† The Au decahedron with 10 {111} and 5 {100} facets is typically composed of 5 tetrahedra that are separated by five equal-angularly twinning planes closed by {221} facets aligned along a 5-fold symmetry axis. The detailed structural analysis of Au decahedral seeds is presented in Fig. S1c, d and S2.† Compared to the {111} and {100} facets, the twin boundaries, top of the 5-fold axis, and tips (corners) of decahedra have higher energy sites due to the lattice strain and lower atomic coordination numbers, favoring the atomic deposition.^13^ Generally, once the Au atoms reduced by ascorbic acid (AA) deposit on these sites with higher surface energy, the migration of adatoms to neighboring facets subsequently occurs *via* surface diffusion. Thus, we performed the synthesis at 25 °C in order to suppress the surface diffusion. Altogether, the Au atoms derived from the reduction of [AuCl_4_]^−^ ions selectively predominate the vertices, tips, or edges of Au decahedral seeds. By optimizing the reduction rate of the [AuCl_4_]^−^ precursor through precisely tailoring the *R* value to avoid symmetry breaking, each kind of site on Au decahedral seeds is able to selectively receive Au atoms at a fixed *R* range, resulting in the formation of the desired penta-twinned NCs.

**Scheme 1:**
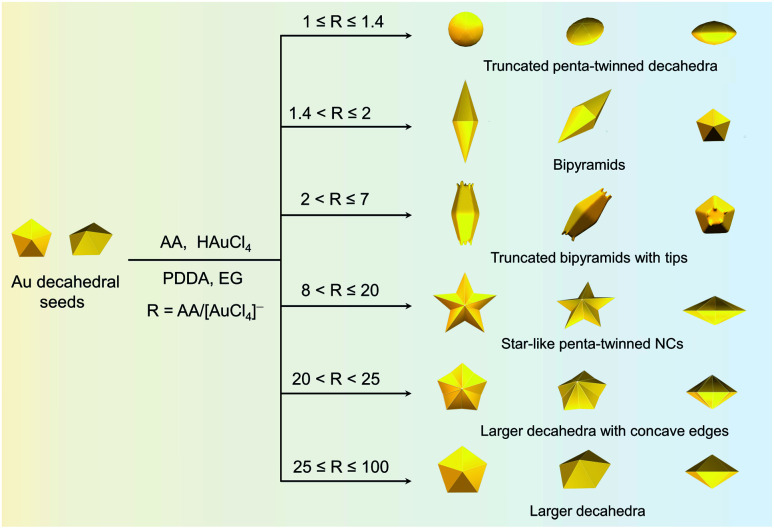
Summary of the experimental *R* values corresponding to the formation of Au penta-twinned nanocrystals with distinctive shapes: truncated penta-twinned decahedra, bipyramids, truncated penta-twinned bipyramids with tips, star-like penta-twinned NCs, decahedra with concave edges, and larger decahedra.

**Fig. 1:**
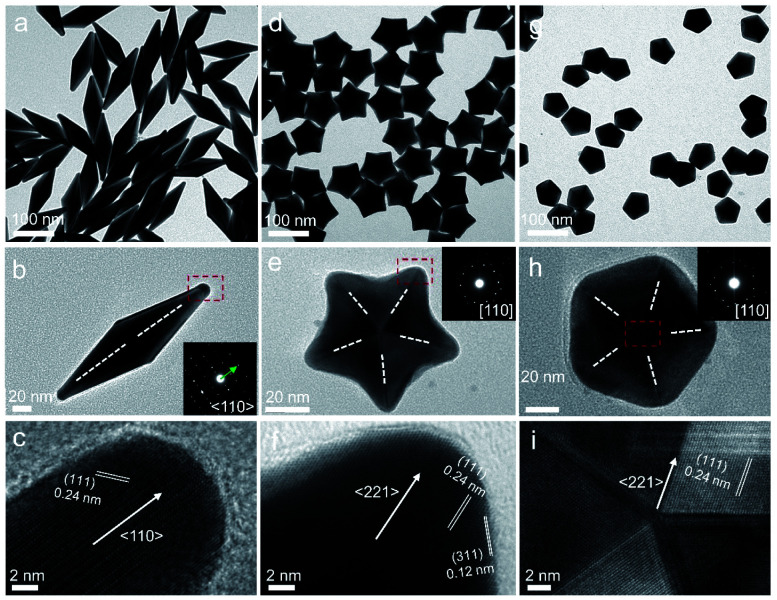
Characterization of penta-twinned NCs prepared using standard protocols: (a and b) TEM and (c) HRTEM images of the Au BPs; (d and e) TEM and (f) HRTEM images of the star-like Au NCs; (g and i) TEM and (i) HRTEM images of the Au DHs. The inset images in (b), (e), and (h) show the corresponding SAED patterns. The white dashed lines and arrows indicate the twin boundaries and the growth direction of Au NCs, respectively.

To demonstrate the formation of different penta-twinned structures by kinetic control, the *R* value was continuously increased from 1 to 100, as shown in Scheme 1. When *R* was in a lower range (1 ≤ *R* ≤ 1.4), the products were dominated by truncated decahedra (Scheme 1 and Fig. S3†). The size of truncated Au decahedra increases from 21.7 ± 0.5, 26.6 ± 0.7, to 29.9 ± 0.8 nm with the *R* increasing from 1.0, 1.2, to 1.4, respectively (Table S1†). As the *R* value increased (1.4 < *R* ≤ 2), the products transformed into truncated BPs with flat {110} facets at both ends aligned along the 5-fold symmetry axis and their aspect ratio also increased from 1.0, 1.4 to 1.5 (Fig. S4 and Table S2†). In this case, the newly formed Au atoms preferentially deposited on Au decahedra and grew along the 〈111〉 direction. We note that even though the truncated Au BPs have high purity above 95%, both ends are flat rather than sharp (Fig. S4†). Generally, compared to the truncated Au BPs, a perfect Au BP shows a stronger localized electronic field at both sharp ends generated from the “antenna effect”.^9^ We thus assumed that if we kept the growth direction along the penta-twinned axis, the ends of truncated Au BPs would become sharp at the cost of more Au atoms, with a stronger localized electronic field at the sharp ends of Au BPs. To verify this concept and the growth-direction-controlling effect of *R*, the *R* value was kept unchanged but the concentration of [AuCl_4_]^−^ was increased to 1 mM. As expected, typical Au BPs (length: 150.1 ± 2.3, width: 47.0 ± 1.5 nm) with sharp ends were synthesized in high yield (Fig. 1a and S5†). The transmission electron microscopy (TEM) and high-resolution TEM (HRTEM) images (Fig. 1b and c) taken from a single Au BP clearly show the twin boundaries. The 0.24 nm interplanar spacing corresponds to the Au {111} facets and the inset selected area electron diffraction (SAED) pattern indicates the corresponding 〈110〉 growth direction (Fig. 1b inset and S6†). When the *R* value reached the range between 2 and 7, the truncated Au BPs had random tips at their corners due to the faster overgrowth (Fig. S7†).

Significantly, an interesting phenomenon that occurred when we further increased the *R* value (8 < *R* ≤ 20) was that the Au decahedral seeds grew into penta-twinned star-like NCs with five identical sharp and twin crystalline arms. This indicated that the growth direction parallel to the 5-fold symmetry axis changed to the one perpendicular to the 5-fold symmetry axis (Fig. 1d–f and S8†). Although many kinds of penta-twinned nanocrystals have been reported in studies, star-like NCs with regular symmetry are still limited due to their thermodynamically unfavorable growth direction. These novel star-like NCs show remarkable monodispersity in terms of both shape and size, and their yield is nearly 100% (Fig. S9†). Further structural analysis using three-dimensional (3D) models displays the highly symmetric star-like Au NCs of our work that could be viewed as stellated polyhedra composed of 20 facets, 40 edges, and 7 vertices (Fig. S10†). We resolved the twin boundaries in the horizontal projection of a single star-like Au NC along the 〈110〉 direction, as highlighted by the white dashed lines (Fig. 1e). The corresponding SAED pattern along its 5-fold axis is the same as that of decahedral Au NCs, also demonstrating that the star-like Au NCs have the same decahedral penta-twinned structure as their seeds (inset in Fig. 1e). HRTEM studies further reveal that each arm of the star-like Au NCs is twin-crystalline, proceeding in the 〈221〉 direction (Fig. 1f). The lattice spacing of 0.24 nm corresponds to the {111} facets. Looking closely, many atomic steps can also be observed, which confirms that the edge surfaces of the five arms are closed by high-index {311} facets with a lattice spacing of 0.12 nm. Interestingly, these structural motifs can be further translated into larger decahedra with concave edges by further increasing the *R* value (20 < *R* < 25), as shown in Fig. S11.† To our surprise, once the *R* value was larger than 25 (25 ≤ *R* ≤ 100), the Au decahedral seeds evolved into typical Au DHs with a larger size of 41.3 ± 2.1 nm (Fig. 1g–i and S12†). These results mean that different growth modes can be achieved to generate a series of well-defined nanostructures in one system just by precisely modulating the *R* value.

### 3.2 Evolution process of penta-twinned Au NCs

**Fig. 2:**
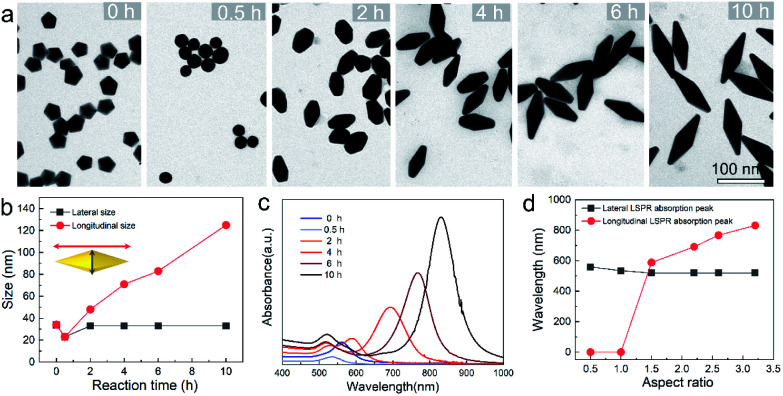
Structure and optical property analysis for the evolution of Au BPs. (a) Typical TEM images of intermediates obtained at different stages: 0, 0.5, 2, 4, 6, and 10 h. (b) Plots of lateral and longitudinal size *versus* reaction time acquired through size analysis. (c) The corresponding UV-vis-NIR spectra. (d) Lateral and longitudinal LSPR maximum *versus* aspect ratios of Au BPs.

To provide a quantitative understanding of the formation of the family of penta-twinned NCs, three typical models including Au BPs, star-like Au NCs, and DHs with distinctly different growth directions were chosen to investigate their growth kinetics. Fig. 2 shows the shape evolution during the synthesis of Au BPs and the change in length and width is plotted *versus* time. Specifically, the 5 twinned-vertices of the Au decahedral seeds were slightly etched to take a round shape at *t* = 0.5 hour (h) due to the initial neutralization reaction between Au^0^ and [AuCl_4_]^−^ at the edges and corners (Fig. 2a). After 2 h of reaction, Au atoms were preferentially deposited on the side surface of the seeds, and thus, the Au decahedra transformed into small truncated Au BPs terminated by Au {110} facets. After another 2 h, the truncated Au BPs gradually elongated along their 5-fold axis. Further extension of the reaction time to 10 h did not cause additional changes to the growth direction, and perfect Au BPs were formed in high yield. We also observed that the horizontal size of the Au BPs was almost consistent with that of the initial decahedral seeds (Fig. 2b), and the longitudinal size increased up to 120.7 ± 2.5, which indicates that the growth prevailed along the 5-fold axis. Fig. 2c displays the corresponding localized surface plasmonic resonance (LSPR) spectra captured at given stages. The longitudinal absorption peak (sensitive to length) gradually redshifted, while the lateral one (ascribed to width) almost remained unchanged as the aspect ratio increased (Fig. 2d). This unique evolution of spectra evidently verifies the observed growth mode displayed in the TEM images. From the penta-twinned structure of the Au BPs, it can be concluded that the Au atoms initially nucleate from both sides of a decahedral seed, followed by gradual growth into an elongated pentagonal bipyramid.

**Fig. 3:**
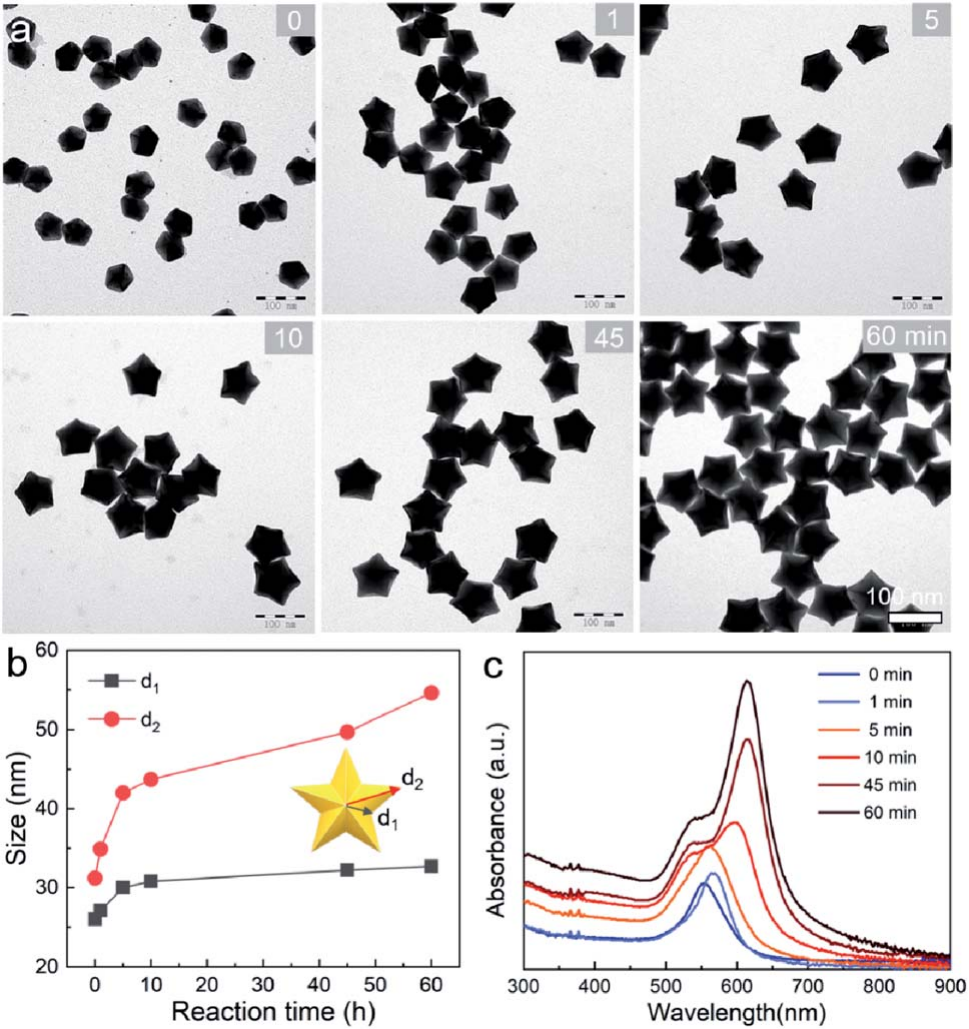
Evolution process of Au NSs. (a and c) TEM images and UV-vis-NIR spectra of Au NSs obtained at different time points: 0, 1, 5, 10, 45, and 60 min. (b) Plots of *d*_1_ and *d*_2_ size *versus* time acquired through size analysis.

We further examined the evolution process of star-like Au NCs, and the *R* value was set as 10 to direct faster reduction kinetics of the metal precursor. TEM images of the products obtained at different stages show that their growth took a pathway distinctly different from that of the Au BPs (Fig. 3a). As the reaction progressed to 1 minute (min), the Au atoms preferentially deposited on the tips of the five twinned boundaries of the decahedral seeds. Here, an abrupt change in the growth direction from vertical to horizontal occurred on the surfaces of crystals at higher *R* values, unlike for those grown at lower *R* values. At *t* = 5 min, more Au atoms were generated and the star-like structure of the NCs became more obvious. The metal tips generally extended along the five twinned boundaries with the increase of time. At a reaction time of 10 min, the majority of the NCs grew into a penta-twinned star-like shape, and further extension of the reaction time to 45 and 60 min caused an obvious increase in the sharpness of the tips along the five twinned boundaries. To quantitatively analyze their structural evolution, *d*_1_ and *d*_2_ of star-like Au NCs were measured at each time point and are summarized in Fig. 3b. The *d*_1_ size displays slight change from 26 ± 1.1 to 32.7 ± 1.6 nm, while the *d*_2_ size continuously becomes longer (from 30.3 ± 0.5 to 56.2 ± 1.3 nm), indicating that Au atoms show a much higher deposition rate on the tips of twin boundaries and a remarkably inhibited diffusion rate. The corresponding UV-vis-NIR spectra of the intermediates agree well with the TEM results: the left peaks change slightly, while the right peak positions redshift and their intensity increases as the reaction proceeds (Fig. 3c). Based on the TEM images, it can be concluded that Au atoms initially deposited onto the tips of the twinned boundaries of the Au decahedral seed instead of depositing on the 5-fold vertex and then grew into star-like Au NCs as the reaction proceeded.

As we did for the case of BPs and star-like Au NCs, we also got insight into the evolution of Au DHs. However, due to the dramatically enhanced reduction kinetics (*R* = 50), once the [AuCl_4_]^−^ precursors were injected, the reduced Au atoms were enriched immediately and were homogeneously deposited on all sites of a decahedral seed instead of selective deposition on the edges and corners. As a result, there was no sufficient time to separate the intermediates from the reaction solution at a very early stage. Fortunately, *in situ* UV-vis-NIR spectroscopy could monitor the changes in crystal size and shape. Fig. S13† displays the corresponding spectra captured at given stages. The shapes of spectra do not obviously change, but their position gradually redshifts, indicating an increase in size. It should be noted that the shape evolution generally depends on the ratio of the growth rate along 〈100〉 and 〈110〉, making it possible to speculate the growth conditions from the geometry.^14^ In the case of decahedra, the overall growth rate of the 〈110〉 direction is 1.4 times higher than that of the 〈100〉 direction (Fig. S2†).

The above experimental results indeed show that once the *R* value is fixed, the growth direction takes a given mode, directing the final morphology of Au NCs. We next verify the authenticity of the structure–*R* value relationship and the general application to growing penta-twinned NCs with different morphologies including Au BPs, star-like NCs, and decahedra. For the synthesis of these NCs with different morphologies, the only variance is the *R* value, which is controlled by varying the concentration of the [AuCl_4_]^−^ precursors. In the case of Au BPs, we set the *R* value as 2. Even though the concentration of the [AuCl_4_]^−^ precursor was tuned from 1.5 to 3 mM, the growth direction still took the 〈110〉 mode. As expected, the morphology of the final products adopted BP shapes with aspect ratios between 2.99 and 3.49, as shown in Fig. S14.† For star-like NCs, the increased *R* value (*R* = 10) results in the growth direction transitioning from the 〈110〉 to 〈221〉 direction. Thus, high-quality star-like NCs were synthesized when the concentration of the [AuCl_4_]^−^ precursor was increased from 0.25 to 1 mM, and the five tips elongated, as summarized in Fig. S15.† Once the *R* value was fixed at 50, the products were dominated by larger decahedra with increasing the concentration of the [AuCl_4_]^−^ precursor from 0.05, 0.1 to 0.2 mM (Fig. S16†).

### 3.3 Elucidation of the growth mechanism

**Scheme 2:**
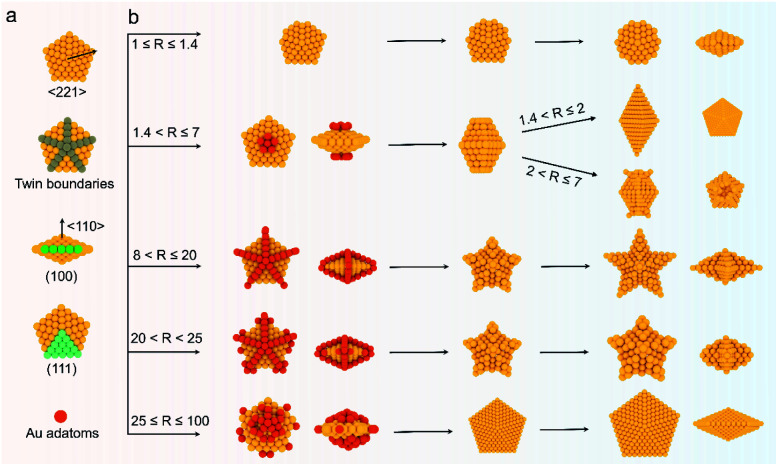
Schematic of the kinetically controlled growth mechanism of different kinds of penta-twinned nanostructures. (a) Structural illustration of Au decahedral seeds. (b) *R* value-driven growth model.

The deposition and diffusion of metal atoms at surfaces play a remarkable role in directing the final crystal morphology. A summary of distinctive growth modes of Au NCs is illustrated in Scheme 2. For a decahedral seed, the top of the 5-fold axis shows the largest surface free energy due to the lowest coordination number and the highest surface strain when compared to twin boundaries, edges, and facets. As a result, the newly generated Au atoms tend to deposit onto the top of the 5-fold axis at lower reduction kinetics, and then the adatoms diffuse to neighboring sites with lower surface free energy, which results in a coexisting growth direction along the 〈110〉 direction on {111} facets.^13^ At a low *R* value (1.4 < *R* ≤ 2), the reduction rate of [AuCl_4_]^−^ precursors is relatively slow, and the adatoms on the top of the 5-fold axis have sufficient time to diffuse to neighboring sites with low surface free energy, forming new {111} facets. Additionally, PDDA as a surfactant can preferentially absorb on Au {111} facets. In the presence of adequate Au atoms, the NCs finally evolve into BPs with sharp apexes along the 〈110〉 direction. As the *R* value increases (2 < *R* ≤ 7), so does the deposition rate, but the main growth direction is still along the 〈110〉 direction in this circumstance. Notably, the reduced nucleation sites at the ends of truncated BPs also contribute to the deposition rate. As such, the newly formed Au atoms prefer to deposit onto these edges and corners that have high surface energy. With the supply of more Au atoms, the truncated BPs evolve into unique truncated BPs with tips at two ends.

Upon increasing *R* to a given value that is higher than 8, a further increased deposition rate would drive newly formed Au atoms to deposit on the tips of the twin boundaries in addition to the top of the 5-fold axis. These five crystal edges of decahedra are actually composed of {100} facets due to the inevitably imperfect growth. Theoretically, the order of surface free energy is {110} > {100} > {111}.^37^ Au adatoms on the tips of twin boundaries thus prefer to diffuse onto {100} facets compared to {111} facets. However, the diffusion rate is very slow because of the lower reaction temperature (25 °C). Additionally, PDDA can suppress the growth along the 〈110〉 direction due to the selective adsorption on Au {111} facets.^38^ Thus, as the reaction time increases, the Au atoms continuously exhibit a high tip deposition/low edge diffusion growth mode, in which the growth should occur selectively on the tips of the twin boundaries along the 〈221〉 direction. Once this circumstance emerges, the growth along the 〈110〉 direction is largely blocked, producing star-like NCs. When the *R* value ranges from 20 to 25, the tip deposition–edge diffusion growth mode still dominates in the main nanostructures. However, the deposition rate of Au atoms is high enough to drive more adatoms to deposit on the {100} edges. As such, the relative growth rate along 〈221〉 slows down and the shape of the nanocrystals at this stage evolves into decahedra with a concave edge. Upon extending the *R* value to be between 25 and 100, the reduction kinetics of Au atoms are greatly enhanced. The increased deposition rate of Au atoms would drive them to either diffuse to or directly nucleate on the edges, making all the decahedra with concave edges convert into typical decahedra. Taken together, the kinetic control based on the *R* value determines the deposition and diffusion behavior of Au atoms on decahedral seeds, thus directing their preferred growth direction along the 〈110〉, 〈100〉, or 〈221〉 directions that define the final structures of NCs.

### 3.4 Fine-tuned plasmonic properties of Au BPs, star-like NCs and DHs

**Fig. 4:**
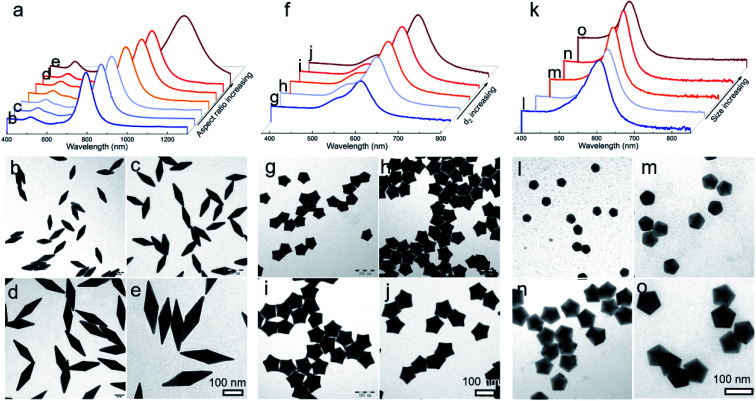
Diversity of products and the corresponding optical properties obtained by this kinetic control method. Effect of seed size on the growth of Au BPs (a–e), NSs (f–j), and decahedra (k–o). (a, f, and k) UV-vis-NIR spectrum and representative TEM images of products obtained by using different seed sizes; all kinds of products were synthesized in one system ((a) *R* = 2, (f) *R* = 10, and (k) *R* = 50).

Given that this well-established mechanism is based on the kinetic principle of crystal growth, this work thus can be extended to be applicable in engineering the size and shape of the desired products. For typical Au BPs, star-like Au NCs, and decahedra, their aspect ratio or size could be readily tuned by simply varying the size of Au decahedral seeds. The results including LSPR spectra and typical TEM images are summarized in Fig. 4. Each kind of product is obtained in a high yield without purification. In the case of Au BPs (*R* = 2), by tailoring the size of Au decahedral seeds (Fig. S17†), the aspect ratio can be tuned from 3.00 to 3.45 (length varied from 85.5 ± 1.9 to 241.2 ± 4.3 nm, equatorial width ranged from 28.4 ± 1.8 to 70.6 ± 2.8 nm) (Fig. 4b–e, S18 and Table S3†), accompanied by the longitudinal LSPR bands changing from 763.1 to 1067.5 nm (Fig. 4a). Currently, there is a great challenge in tuning the LSPR peak position in an extremely broad region from the visible to second near-infrared (NIR-II) region (1000–1350 nm, owing to the built-in advantages of deeper tissue penetration capability and less energy loss in biomedicine).^20,29^ Given the discussion above, the precisely tuned plasmonic properties of Au BPs are expected to be used in NIR-induced hot electron excitation, surface-enhanced spectroscopy, and biomedicine. Moreover, the high quality of Au BPs allows us to get the relationship between longitudinal LSPR peak position and length. Fig. S19† shows the linear calibration plots of longitudinal LSPR peak positions *versus* the length of Au BPs, where the *Y* value represents the peak position, while the *X* value represents the longitudinal size. The *r*^2^ (*r* refers to relation coefficient) of the linear regression equation is 0.9994, extremely close to 1, indicating a good linear relationship. Additionally, as an emerging example, star-like Au NCs with regular symmetry have rarely been reported to date, especially those with finely tuned shapes, sizes, and plasmonic properties. Here, just by simply controlling the size of Au decahedral seeds, we can achieve high-quality star-like Au NCs with different *d*_2_ sizes varying from 30.3 ± 0.5 to 56.2 ± 1.3 nm (Fig. 4f–j, S20 and Table S4†). The corresponding LSPR spectra were also recorded, with the plasmon band red shifting from 477.2 to 613.3 nm, as the size of seeds increased. Similarly, for Au decahedral seed sizes between 19.1 ± 1.2 and 38.7 ± 1.3, larger Au decahedra were obtained with controlled edge sizes from 23.2 ± 1.1 to 63.2 ± 2.0 nm, accompanied by a much narrower LSPR position ranging from 455.1 to 536.0 nm, as shown in Fig. 4k–o, S21 and Table S5.†

### 3.5 Universality for the growth of a second metal

**Fig. 5:**
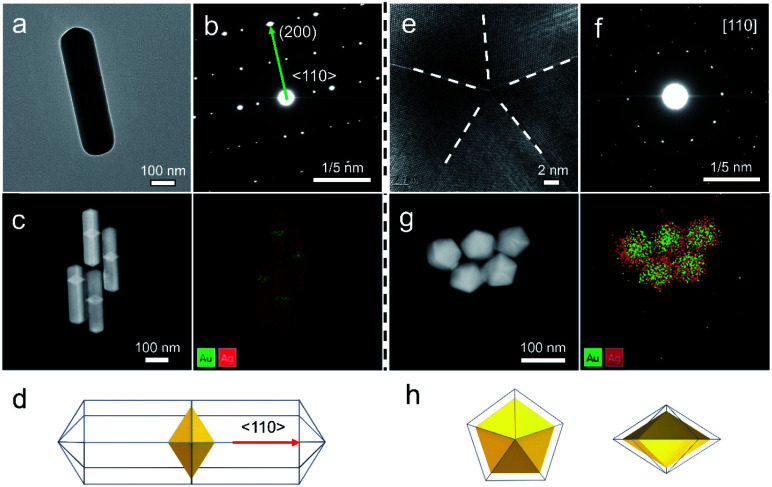
Universality for the synthesis of Au@Ag NRs and DHs. (a and e) TEM images of Au@Ag NRs and DHs produced through the standard growth process, at a given *R* value of 2 and 50, respectively. (b and f) The corresponding SAED patterns. (c and g) HAADF-STEM and elemental maps. (d and h) The corresponding schemes of Au@Ag NRs and DHs.

Our strategy can be further applied to the growth of a second metal on Au decahedral seeds and achieve the visualization of seeds in the final products, offering an opportunity to get insight into the growth mechanism of heterogeneous penta-twinned NCs. We chose Ag as the second metal, with a low atomic mass (107.86) compared to Au (196.96), which ensures clear contrast and detailed visualization in the HAADF-STEM mode. Moreover, Ag shows extremely smaller lattice mismatch with Au, facilitating epitaxial growth and preserving the crystallinity of the original Au seed. As expected, when the *R* value was set as 2, Au@Ag NRs were synthesized with atomic ratios of 93% for Au and 7% for Ag (Fig. 5a–d). It can be clearly observed that Au decahedral seeds are located in the center of the NRs and that the Ag grew on seeds along the 5-fold axis (Fig. 5c). Moreover, the aspect ratio of Au@Ag NRs can be readily tuned from 1.2 to 3.9 (Fig. S22a–c†), accompanied by the LSPR peak position increasing from 507.6 to 1260.2 nm (Fig. S22d and e†). It should be noted that the longitudinal size of Au@Ag NRs shows a good linear relationship with the dipolar lateral resonance peak positions, which gives us an advantageous opportunity to precisely engineer their LSPR properties in a wide range from the visible to NIR-II region. Once the *R* value was increased to 50, the growth direction of Ag atoms on Au decahedral seeds changed from vertical to horizontal, resulting in typically larger Au@Ag DHs (Fig. 5e–h). Taken together, it can be reasonably envisioned that the penta-twinned nanostructures with heterometals might be fabricated by finely modulating the growth direction of the second or third species.

## 4 Conclusions

In summary, we highlight that the family of penta-twinned Au NCs can be synthesized in a high yield (more than 95%) by a kinetically-controlled seeded method in one growth system. In our case, modifications in the deposition kinetics of atoms can be achieved *via* quantitative control of the *R* value (ratio of reductant/Au precursor), directing whether horizontal or vertical features along the 5-fold axis of decahedral seeds are produced at specific seed locations. The use of this kind of kinetic control can result in seven kinds of penta-twinned NCs with tunable sizes and high purity in one system, which are usually obtained under different growth conditions. The ultrahigh structural precision in shape, size, and high product yield enable the fine-tuning of plasmonic optical responses from penta-twinned NCs, particularly for Au BPs with LSPR bands ranging from visible to NIR-II. Moreover, the horizontal or vertical growth of a second metal (silver) on penta-twinned gold seeds can be reached through minor modification of *R*, which opens a new avenue of mechanistic investigation by visualizing the seed localization within the final particles. This present work demonstrates a general paradigm for the kinetically modulated growth of penta-twinned Au crystals in a controllable and predictable manner and could be extended to the synthesis of other families of NCs.

## Author contributions

T. Z., C. L., and Y. L. designed the project; T. Z. performed the synthesis, characterization, and analysis; X. L. and Y. S. draw the schematic diagram; C. L. and Y. L. supervised the research; T. Z. wrote the manuscript. All the authors analyzed the data, discussed the results, and commented on the manuscript.

## Conflicts of interest

There are no conflicts to declare.

## Supplementary Material

SC-012-D1SC03040J-s001
