# Supplementary material for: A universal route with fine kinetic control to a family of penta-twinned gold nanocrystals
Source: Chem Sci. 2021 Aug 24;12(38):12631–9. doi: 10.1039/d1sc03040j (PMC8494040; doi:10.1039/d1sc03040j)
Supplement: SC-012-D1SC03040J-s001 [file SC-012-D1SC03040J-s001.pdf]

Electronic Supplementary Material (ESI) for Chemical Science.
This journal is © The Royal Society of Chemistry 2021

Supporting Information

A Universal Route with Finely Kinetical Control to the Family of Penta-twinned Gold Nanocrystals

Tao Zhang,[a] Xuejiao Li,[a,c] Yiqiang Sun,[b] Dilong Liu,[a] Cuncheng Li,*[b] Weiping Cai,[a] and Yue Li*[a]

[a] *Key Laboratory of Materials Physics, Institute of Solid State Physics, HFIPS, Chinese Academy of Sciences, Hefei 230031, China*

[b] *School of Chemistry and Chemical Engineering, University of Jinan, Jinan 250022, Shandong, P. R. China*

[c] *University of Science and Technology of China, Hefei, 230026, P. R. China*

*Corresponding author. *E-mail:* chm_licc@ujn.edu.cn, yueli@issp.ac.cn

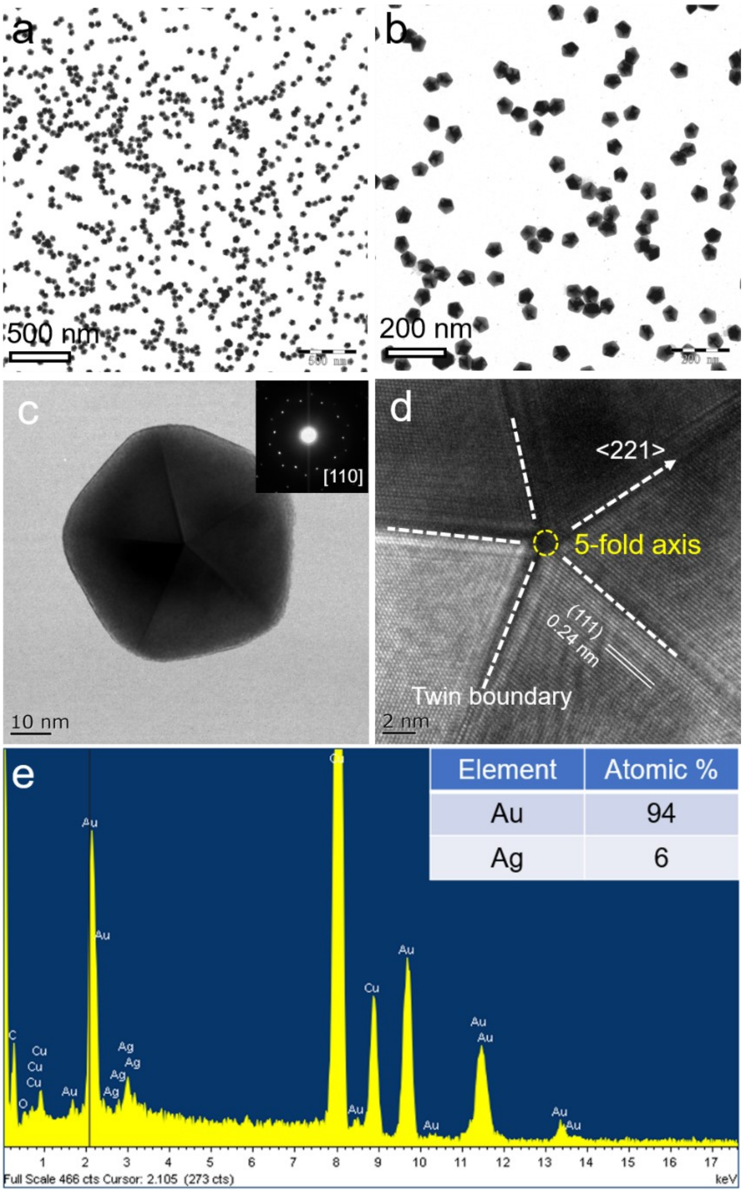

Figure S1. Characterization of Au decahedral seeds. (a, b) Low- and (c) high-magnification TEM images. (d) HRTEM image. (e) The corresponding EDS spectrum. The inset image in (c) shows the corresponding SAED pattern. The white dashed lines and yellow circle in (d) indicate the twin boundaries and 5-fold axis of decahedral seed, respectively.

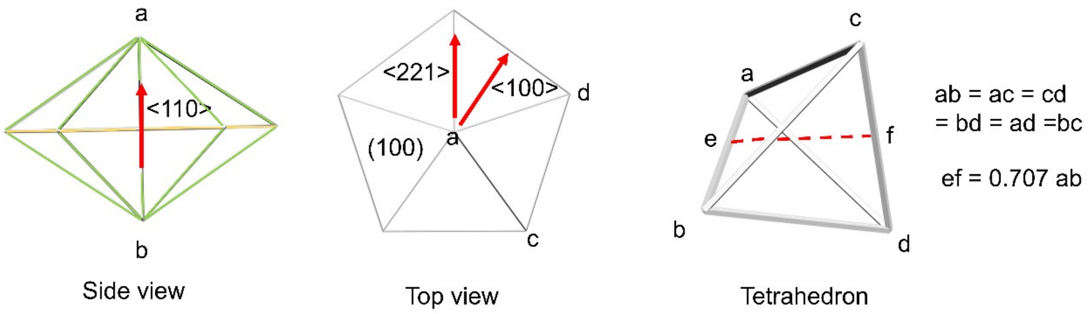

Figure S2. Geometric scheme of Au decahedra. Au decahedron with 10 {111} and 5{100} facets is typically composed of 5 tetrahedrons that are separated by five equal-angularly twinning planes closed by {211} facets aligned along 5-fold symmetry axis. The red arrow indicates the <221> direction. The green and yellow lines indicate {100} and {211} facets, respectively. The length of ef is equal to 0.707 times shorter than that of ab.

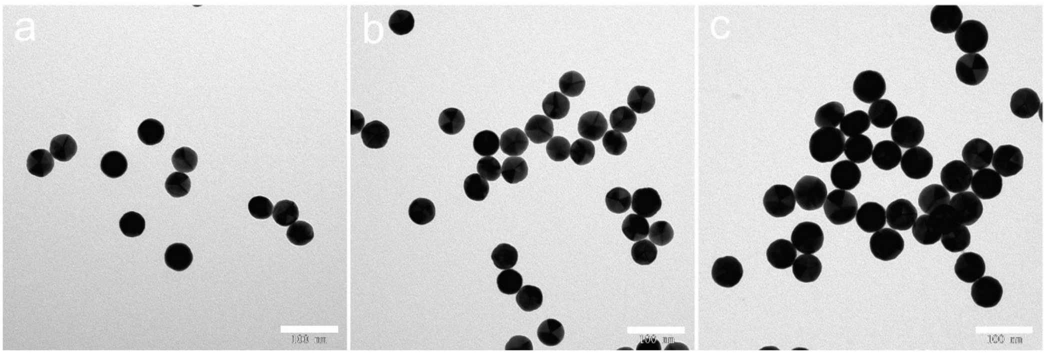

Figure S3. TEM images of typical truncated penta-twinned decahedra obtained at (a) R=1.0, (b) R=1.2, and (c) R=1.4, respectively. All scale bars are 100 nm.

Table S1. The size (d) distribution of the corresponding truncated Au decahedra in Figure S3.

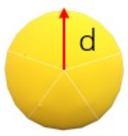

R value	d (nm)
1.0	21.7±0.5
1.2	26.6±0.7
1.4	29.9±0.8

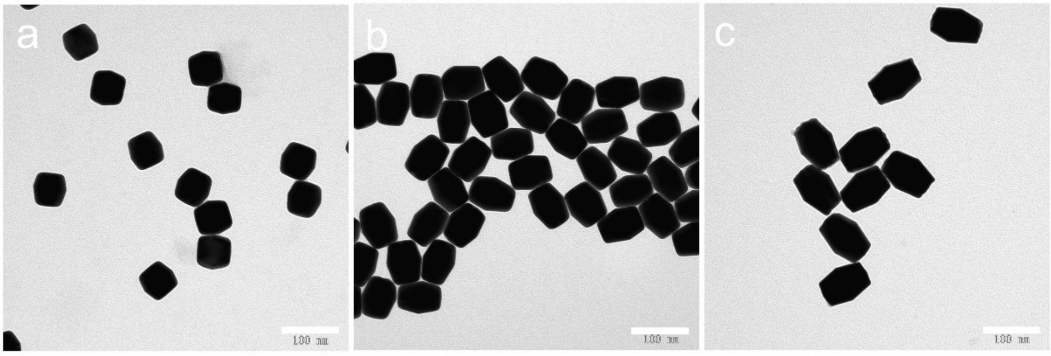

Figure S4. TEM images of the typical truncated Au BPs obtained at (a) R=1.6, (b) R=1.8, and (c) R=2. In this case, the concentration of Au precursor was 0.5 mM, while the concentration of AA increased from 0.8, 0.9 to 1.0 mM, respectively. All scale bars are 100 nm.

Table S2. Dimensions (length and width) and aspect ratios of the corresponding truncated Au BPs in Figure S4.

Length (nm)	width (nm)	Aspect ratio
59.1±1.2	59.0±0.7	1.0
72.9±1.8	52.6±0.5	1.4
88.6±2.0	59.6±0.9	1.5

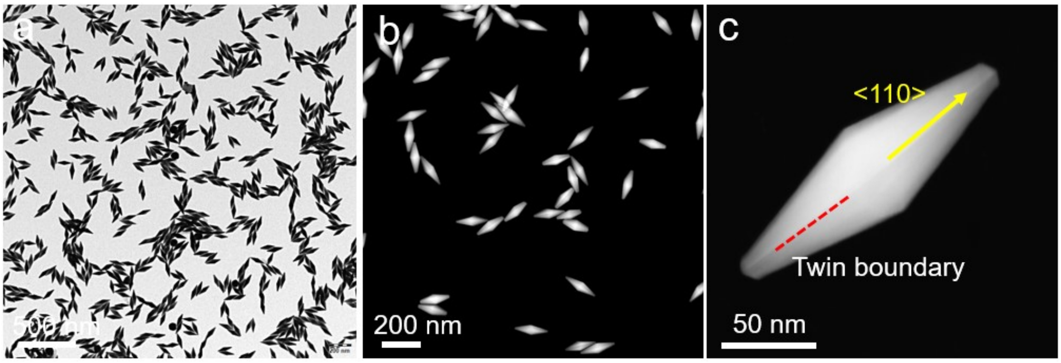

Figure S5. TEM (a) and HAADF-STEM (b, c) images of the typical Au BPs obtained at R=2. The red dash line and yellow arrow indicate the twin boundary and growth direction, respectively.

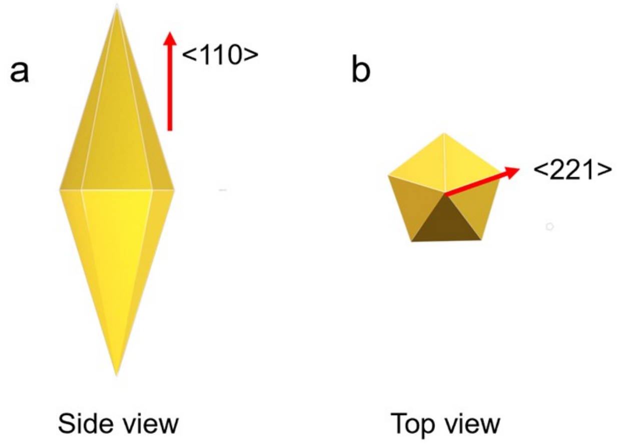

Figure S6. 3D models of Au BPs. The red arrows indicate the growth directions.

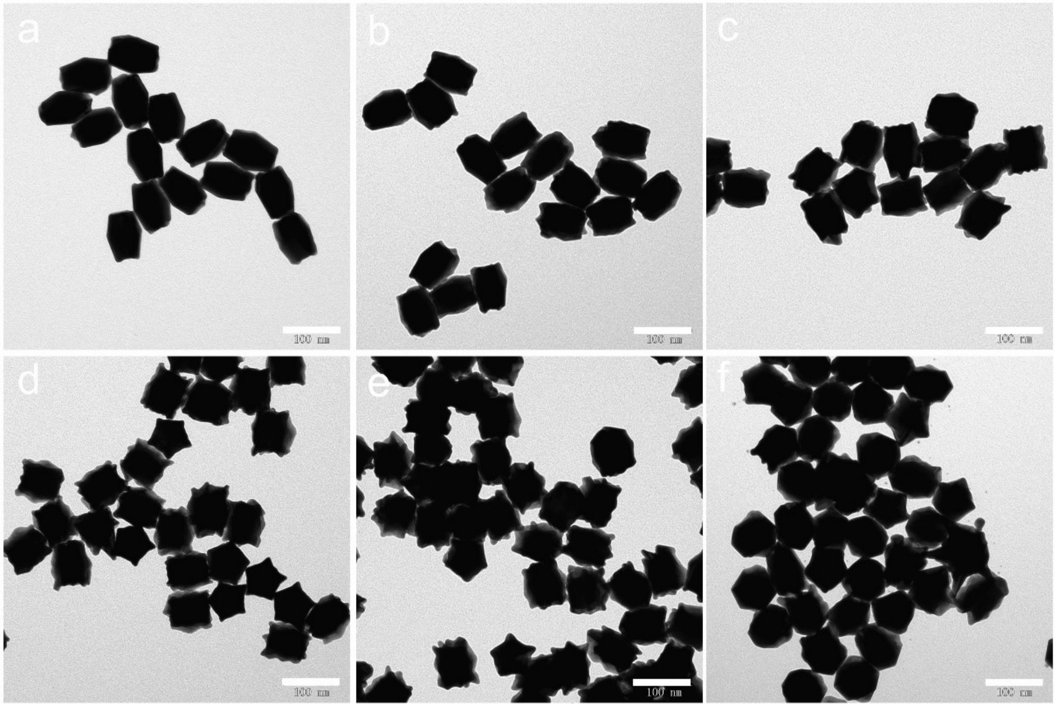

Figure S7. TEM images of typical truncated Au BPs with tips obtained at (a) R=2.5, (b) R=3, (c) R=4, (d) R=5, (e) R=6, and (f) R=7. In this case, the concentration of Au precursor was 0.5 mM, while the concentration of AA increased from 1.25, 1.5, 2.0, 2.5, 3.0 to 3.5 mM, respectively. All scale bars are 100 nm.

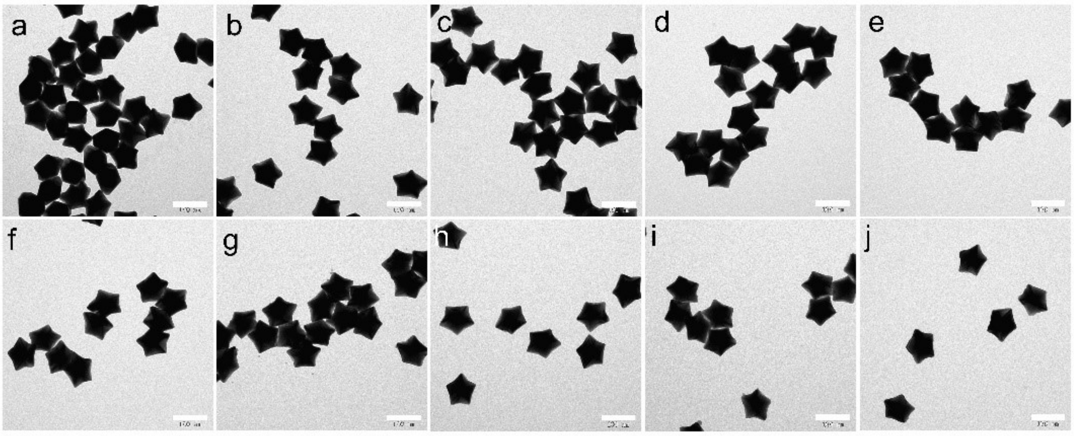

Figure S8. TEM images of star-like Au NCs obtained at (a) R=8, (b) R=9, (c) R=10, (d) R=11, (e) R=12, (f) R=13, (g) R=14, (h) R=15, (i) R=16, and (j) R=20. In this case, the concentration of Au precursor was 0.5 mM, while the concentration of AA increased from 4.0, 4.5, 5.0, 5.5, 6.0, 6.5, 7.0, 7.5, 8.0 to 10 mM, respectively. All scale bars are 100 nm.

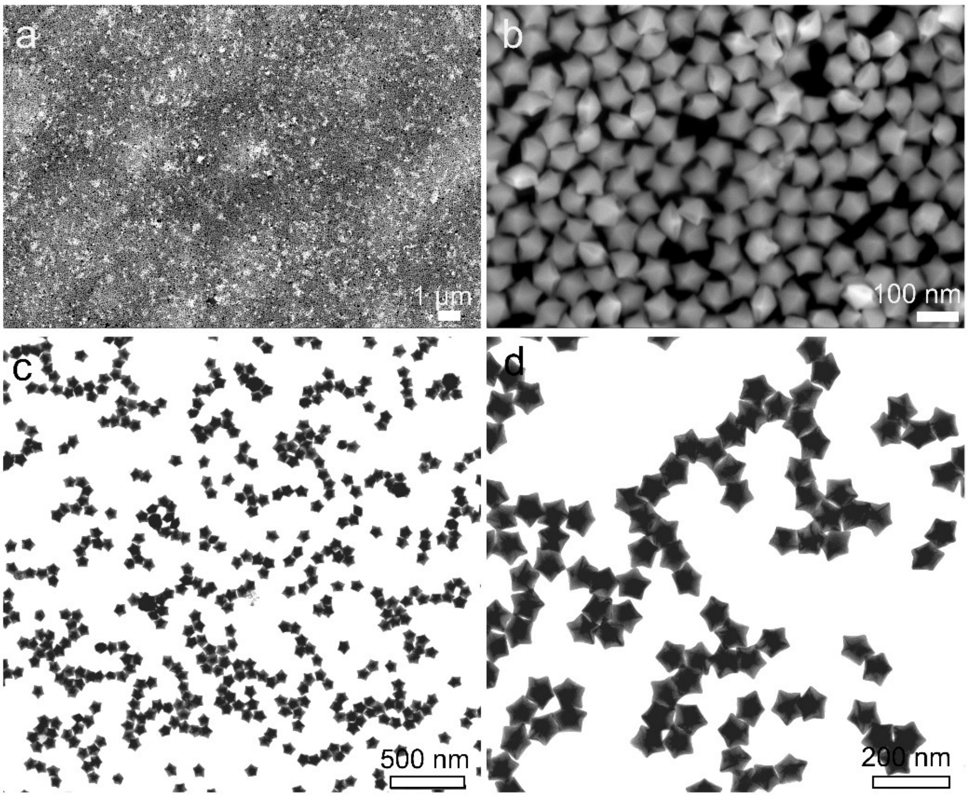

Figure S9. (a-b) Low- and high-resolution SEM images. (c-d) Low- and high-resolution TEM images.

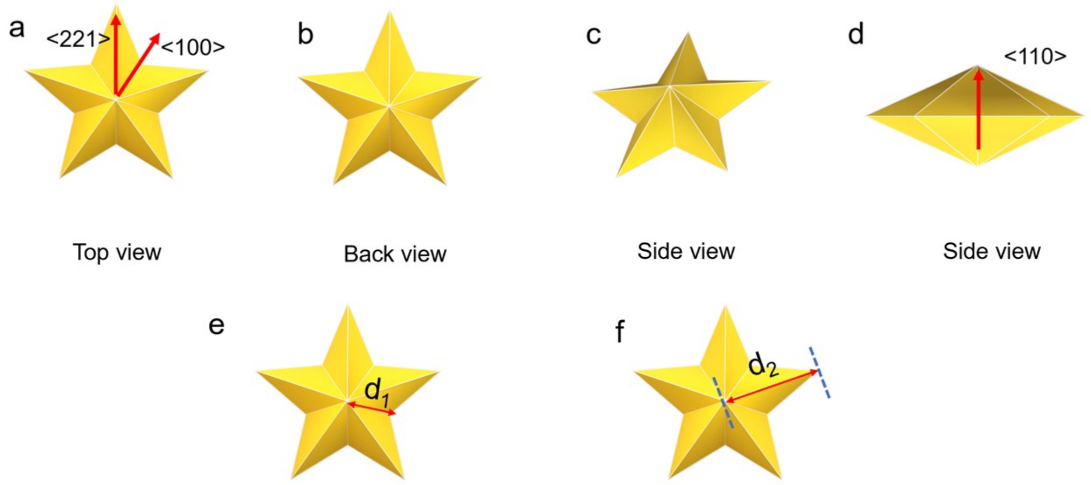

Figure S10. 3D models of the highly symmetric star-like Au NCs, which be viewed as a stellated polyhedron composed of 20 facets, 40 edges, and 7 vertices. The red arrows indicate the growth directions. The d_1 and d_2 indicate the projected length of axis to edge and axis to tip, respectively.

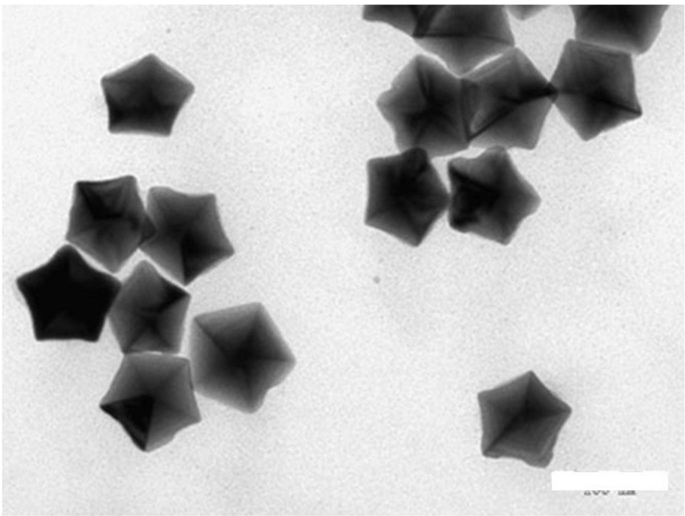

Figure S11. TEM image of Au decahedra with concave edges synthesized at R=24. Scale bar is 100 nm.

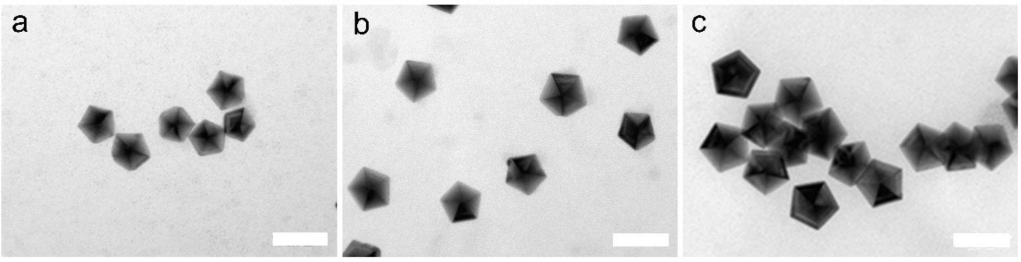

Figure S12. TEM images of Au decahedra obtained at (a) R=100, (b) R=50, and (c) R=25. In this case, the concentration of AA precursor was 5 mM, while the concentration of AA increased from 0.05, 0.1 to 0.2 mM, respectively. All scale bars are 100 nm.

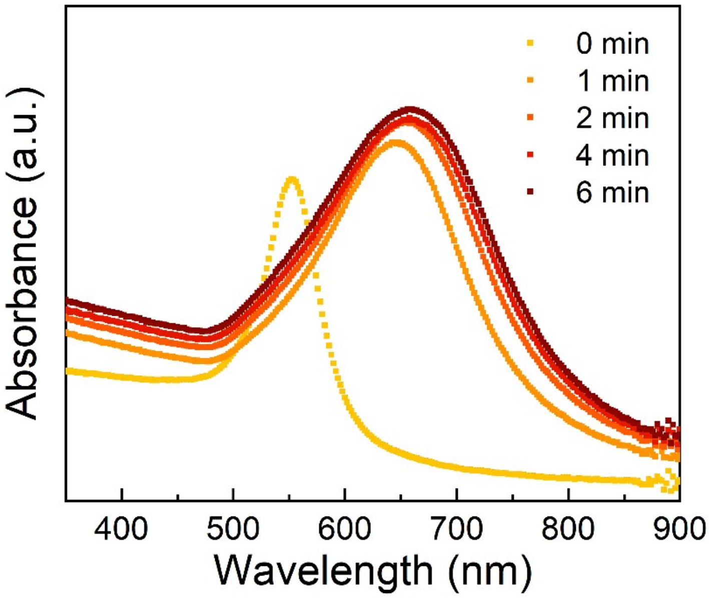

Figure S13. UV-vis-NIR spectra of Au DHs obtained at different time points: 0, 1, 2, 4, and 6 min.

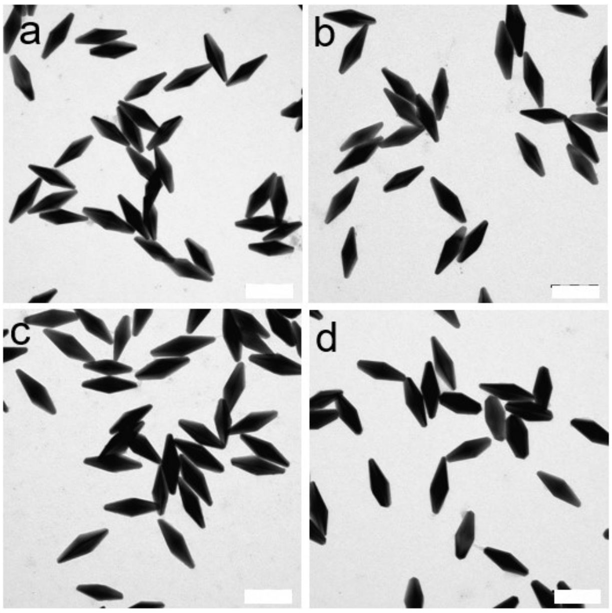

Figure S14. Au BPs synthesized at a fixed R value of 2. The concentration of AA was (a) 1.5, (b) 2, (c) 2.5, and (d) 3 mM, while the concentration of $[AuCl_4]^-$ was set as 0.75, 1, 1.25, and 1.5 mM, respectively. The corresponding aspect ratio is 2.99, 3.18, 3.49, and 3.26, respectively. All scale bars are 100 nm.

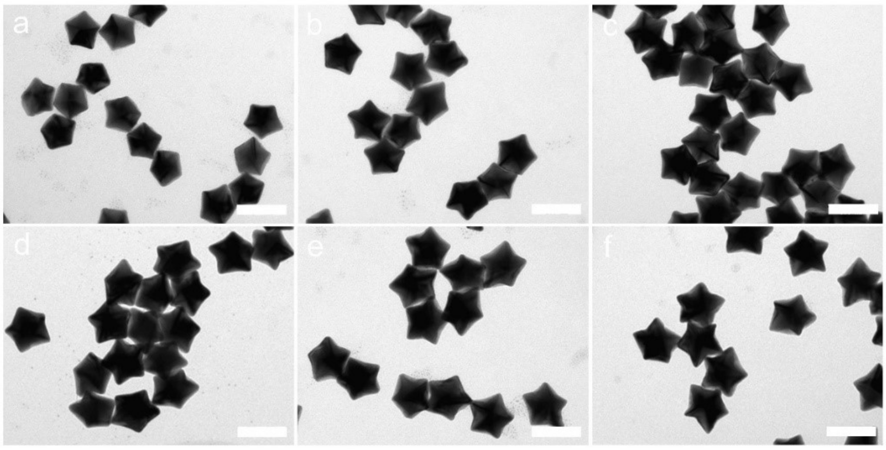

Figure S15. Star-like Au NCs synthesized at a fixed R value of 10. The concentration of AA was (a) 2.5, (b) 3.75, (c) 5, (d) 6.25, (e) 7.5, and (f) 10 mM, while the concentration of $[AuCl_4]^-$ was set as 0.25, 0.375, 0.5, 0.625, 0.75, and 1 mM, respectively. The d_1 is 34 ± 0.5, 38.5 ± 0.7, 41.2 ± 0.5, 46.7 ± 0.6, 48.3 ± 0.9, 54.1 ± 1.5 nm, respectively.

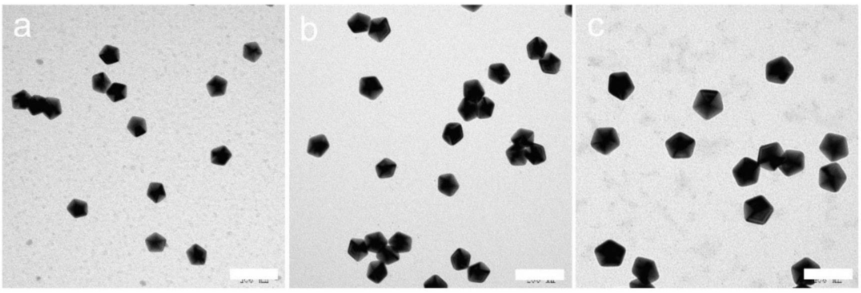

Figure S16. Au decahedra obtained at a given R value of 50. The edge size of decahedra became larger with increasing the concentration of $[AuCl_4]^-$ precursor from (a) 0.05, (b) 0.1 to (c) 0.2 mM. The edge size is 25.4 ± 0.6, 28.0 ± 0.7, and 37.5 ± 0.9, respectively. All scale bars are 100 nm.

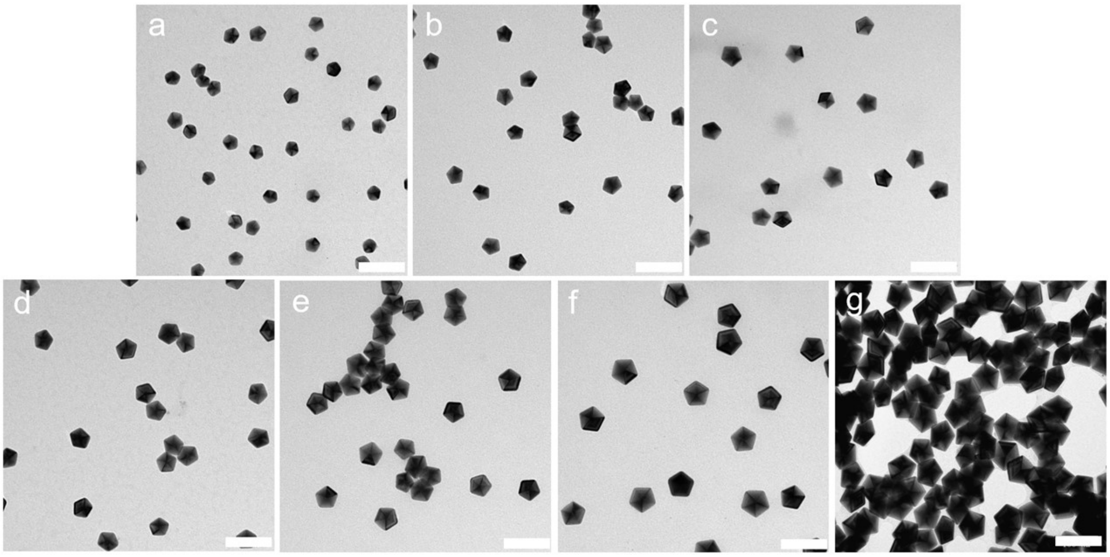

Figure S17. TEM images of Au decahedral seeds with different average edge length: (a) 19.1±1.2, (b) 23.3±2.0, (c) 25.0±2.2, (d) 28.5±1.8, (e) 32.2±1.1, (f) 38.7±1.3, and (g) 49.6±1.8 nm. All scale bars are 50 nm.

The size of Au decahedral seeds is increased with the decrease of reaction temperature. Au decahedral seeds with average edge length of 19.1±1.2, 23.3±2.0, 25.0±2.2, 28.5±1.8, 32.2±1.1, 38.7±1.3, and 49.6±1.8 nm could be obtained by only

changing the reaction temperature as 220, 218, 215, 210, 204, 200, and 185 °C, respectively.

(a)-(f) were used for the growth of Au BPs in Figure 5a. (a), (b), (c), (e), and (f) were used for the synthesis of star-like Au NCs in Figure 5f. (a), (c), (d), (e), and (f) were used for the growth of Au decahedra with larger size.

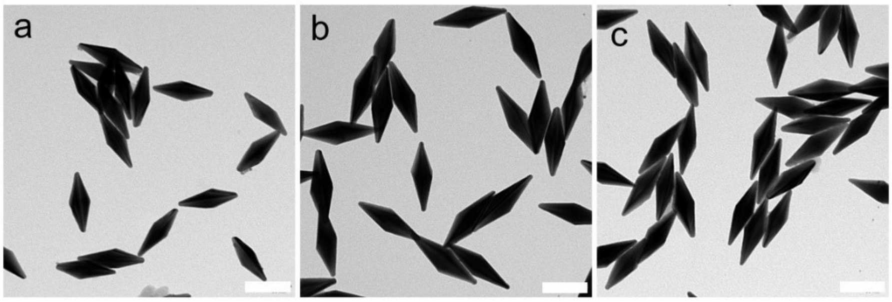

Figure S18. TEM images (a), (b), and (c) of Au BPs with the aspect ratio of 3.17, 3.19, and 3.26, respectively, obtained from (c), (d), and (e) in Figure S9. All scale bars are 100 nm.

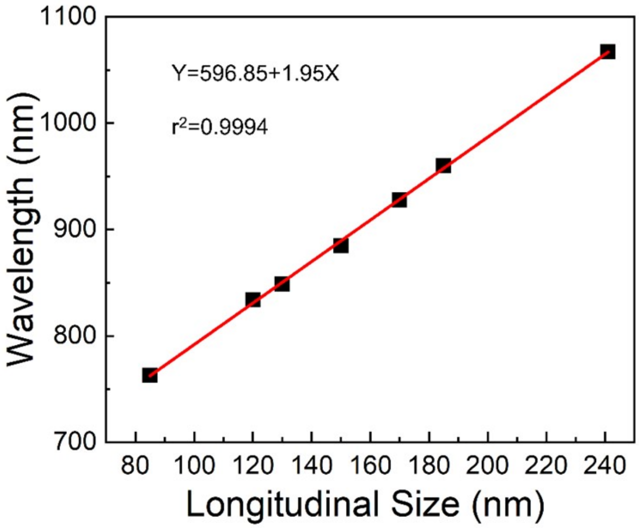

Figure S19. Plot for the longitudinal LSPR peak positions as a function of longitudinal sizes of Au BPs. The Y value presents peak position, while the X value represents the longitudinal size. The r^2 of linear regression equations is 0.9994, extremely close to 1, indicating a well linear relationship.

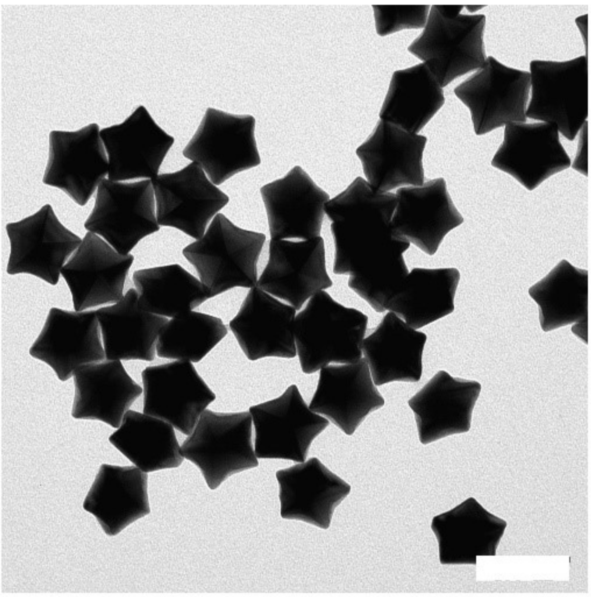

Figure S20. TEM image of star-like Au NCs with the d_2 size of 40.1±2.5 nm, obtained from (d) in Figure S9. Scale bar is 100 nm.

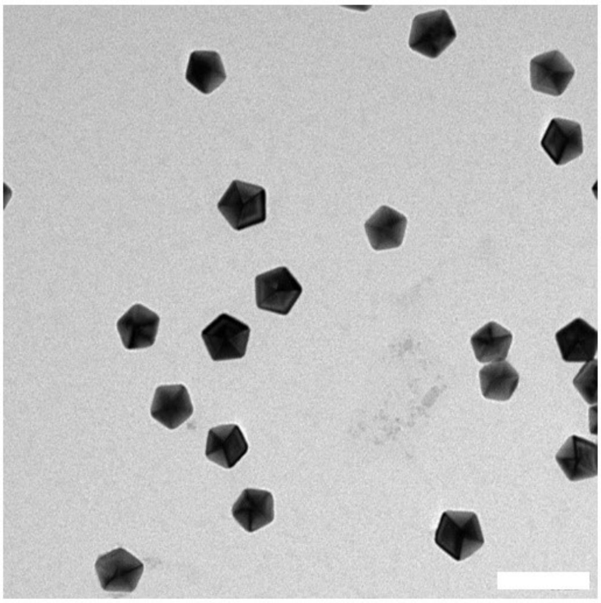

Figure S21. TEM image of Au decahedra with the edge length of 29.4±1.8 nm, obtained from (b) in Figure S9. Scale bar is 100 nm.

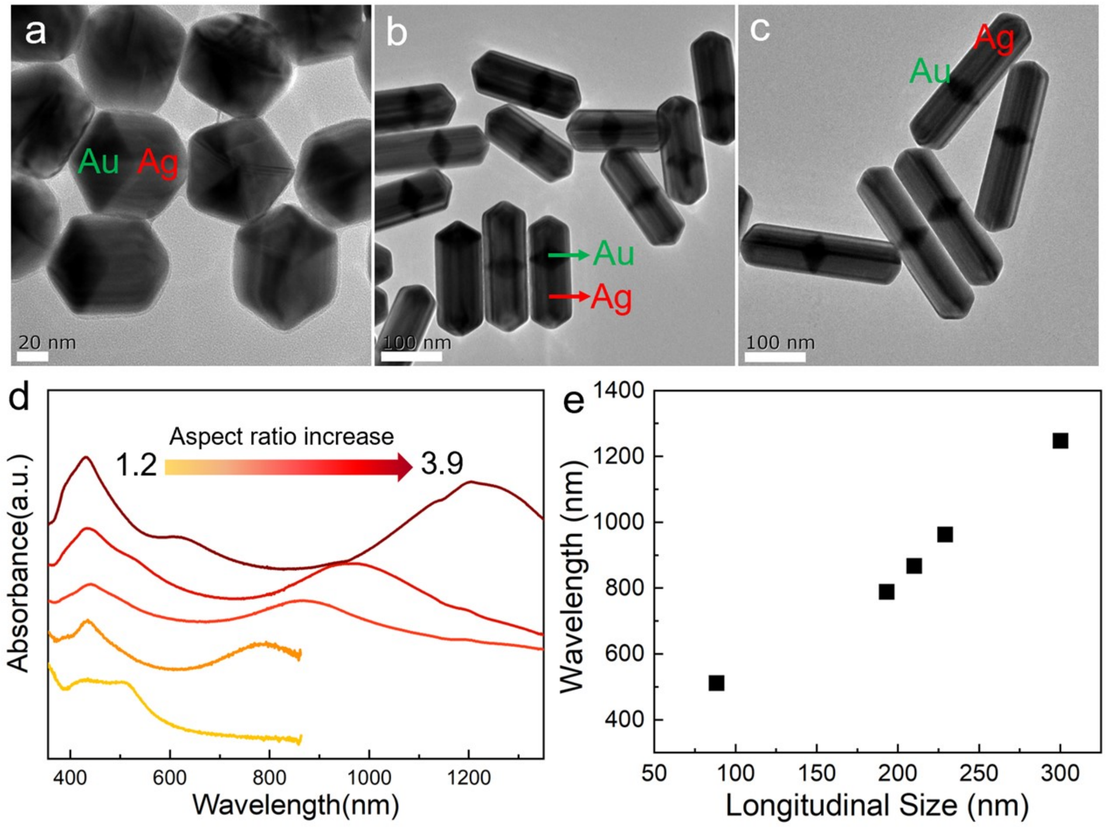

Figure S22. Size tunability of Au@Ag NRs. (a-c) TEM images of presentive Au@Ag NRs with longitudinal size of 88.2±3.1, 194.5±5.2, and 301.1±6.8 nm, respectively. The dark Au core and light Ag shell are marked by green and yellow, respectively. (d) UV-vis-NIR spectrum of Au@Ag NRs with aspect ratio range from 1.2 to 3.9. (e) Plot for the longitudinal LSPR peak positions as a function of longitudinal sizes.

Table S3. Dimensions (length and width), aspect ratios, and LSPR of the Au BPs prepared using different Au decahedral seeds.

Seed Size (nm)	Length (nm)	width (nm)	Aspect ratio	LSPR
19.1±1.2	85.5±1.9	28.4±1.8	3.00	763.1
23.3±2.0	120.7±2.5	38.2±1.9	3.14	834.3
25.0±2.2	130.4±3.1	41.1±2.6	3.17	849.0
28.5±1.8	150.1±2.3	47.0±1.5	3.19	885.7
32.2±1.1	170.8±2.1	52.2±2.0	3.26	928.5
38.7±1.3	185.1±2.7	55.3±1.7	3.36	960.1
49.6±1.8	241.2±4.3	70.6±2.8	3.45	1067.5

Table S4. Dimensions (d_2) and LSPR of the star-like Au NCs prepared using different Au decahedral seeds.

Seed Size (nm)	d_2 (nm)	LSPR
19.1±1.2	30.3±0.5	477.2
23.3±2.0	36.2±1.6	515.8
25.0±2.2	40.1±2.5	541.8
32.2±1.1	49.6±1.9	575.3
38.7±1.3	56.2±1.3	613.3

Table S5. Dimensions (edge size) and LSPR of the Au decahedrons prepared using different Au decahedral seeds.

Seed Size (nm)	Edge length (nm)	LSPR
19.1±1.2	23.2±1.1	455.1
25.0±2.2	29.4±1.8	476.5
28.5±1.8	41.3±2.1	491.8
32.2±1.1	50.7±1.9	520.8
38.7±1.3	63.2±2.0	536.0
